# Differences in the constituent fiber types contribute to the intermuscular variation in the timing of the developmental synapse elimination

**DOI:** 10.1038/s41598-019-45090-6

**Published:** 2019-06-18

**Authors:** Young il Lee

**Affiliations:** Department of Biology, Texas A&M University, College Station, TX 77843 Texas, USA

**Keywords:** Synaptic development, Cell biology

## Abstract

The emergence of a mature nervous system requires a significant refinement of the synaptic connections initially formed during development. Redundant synaptic connections are removed in a process known as synapse elimination. Synapse elimination has been extensively studied at the rodent neuromuscular junction (NMJ). Although several axons initially converge onto each postsynaptic muscle fiber, all redundant inputs are removed during early postnatal development until a single motor neuron innervates each NMJ. Neuronal activity as well as synaptic glia influence the course of synapse elimination. It is, however, unclear whether target muscle fibers are more than naïve substrates in this process. I examined the influence of target myofiber contractile properties on synapse elimination. The timing of redundant input removal in muscles examined correlates strongly with their proportion of slow myofibers: muscles with more slow fibers undergo elimination more slowly. Moreover, this intermuscular difference in the timing of synapse elimination appears to result from local differences in the rate of elimination on fast versus slow myofibers. These results, therefore, imply that differences in the constituent fiber types help account for the variation in the timing of the developmental synapse elimination between muscles and show that the muscle plays a role in the process.

## Introduction

Synaptic connections – and the neuronal networks which they form – undergo significant transformation during maturation of the nervous system. Such maturation is essential for proper architecture and function of the nervous system. This synaptic transformation includes synapse elimination, a process in which multiple immature presynaptic inputs converge at and compete for control of a common postsynaptic target. Similar pruning occurs throughout developing nervous systems^1,2^. In the central nervous system (CNS), pruning contributes to learning and memory^3,4^. The importance of proper synaptic maturation is highlighted by the growing body of literature that implicates defects in developmental CNS synapse elimination in the genesis of debilitating neurodevelopmental disorders^2,5,6^.

Although widespread throughout the developing nervous system, synapse elimination is most extensively studied and perhaps best understood at developing rodent neuromuscular junctions (NMJs), cholinergic synaptic connections between axon terminals of spinal motor neurons and their target skeletal muscle fibers. At each mature NMJ, the presynaptic terminals of a single motor neuron appose high-density aggregates of postsynaptic acetylcholine receptors (AChRs), and the processes of terminal Schwann cells (tSCs) cap the synaptic apposition. In rodents, this is achieved within the first three postnatal weeks by locally pruning all but one of up to ~10 motor axons that converge onto a postsynaptic muscle fiber^7–9^. Synapse elimination at developing endplates, like elsewhere in the nervous system^10–13^, is influenced by activity. The relative levels and the pattern of activity influence the winner and the timing with which the competition amongst immature axonal inputs that converge at developing endplates is resolved^14–17^. In addition, recent studies also implicate active glial participation in the appropriate and timely pruning of excess axonal inputs^18–21^. Little is known about what instructive role, if any, the postsynaptic targets play in synapse elimination.

Based on the two fundamentally different types of contractions they produce, skeletal muscle fibers can be classified either as “tonic” (lacking action potential activity and generating slow and graded contractions) or “twitch” (generating both action potential and twitch contractions)^22,23^. Tonic fibers are common in amphibians and reptiles but found only in a subset of extraocular muscles in mammals^24–29^. In all species examined, these tonic fibers remain innervated by multiple motor axons into adulthood^29–33^. In contrast, the twitch fibers achieve single innervation. The twitch fibers are further divided, crudely, into two sub-classes based on their contraction speeds: slow twitch (type I) and fast twitch (type II)^34^. Such divergence between tonic and twitch fibers, in their ability and/or need to tolerate multiple motor innervation, hints that properties of these two distinct twitch fibers types (contractile or otherwise) may also differentially influence excess motor input removal.

The interaction between the nerve and muscle at NMJs presents an added layer of complexity in determining the contributions of each during synapse elimination. The contractile properties of the slow and fast fibers match the activity patterns of their respective presynaptic motor neurons. The presynaptic nerve terminals and the target muscle fibers influence each other via an exchange of signals known to occur at NMJs (e.g.^35–39^). Cross-innervation experiments – in which muscles are experimentally innervated by foreign nerves that normally innervate muscle of different fiber type – clearly show muscle fiber types are sensitive to the activity patterns presented via the innervating motor axons. These fibers alter their contractile properties to match the firing patterns of the axons present in the foreign nerve^40,41^. The normal differentiation of fiber types and their normal intramuscular distribution, however, can occur even in the absence of innervation^42^. Thus, it may be difficult to discern whether fiber type-specific differences in synapse elimination stem from muscle-autonomous influences and/or as nerve-driven changes to the postsynaptic fiber types.

The aim of this study was to examine, with the use of imaging and genetic tools currently available, whether innate properties of the developing postsynaptic muscle fibers actively influence the process of neuromuscular synapse elimination. I report that synapse elimination is delayed for developing NMJs situated on slow fibers compared to those on fast fibers. Moreover, a muscle fiber-specific mutation that reduces the fraction of type I fibers hastens synapse elimination. My current findings, thus, strongly suggest that target muscle fiber type influences motor axon input pruning during developmental synapse elimination.

## Methods

### Animals

Experiments were conducted in accordance with National Institutes of Health guidelines and were approved by the Institutional Animal Care and Use Committees at Texas A&M University. The intramuscular comparison of synapse elimination for NMJs situated on slow and fast fibers was performed in wildtype C57BL/6 mice. All mutants and transgenic animals utilized have the same C57BL/6 genetic background. The transgenic overexpression of PGC1α was achieved under a muscle-specific muscle creatine kinase promoter (MCK-PGC1α; JAX 008231). Mice whose skeletal muscle fibers lacked PGC1α were generated by breeding animals that harbor floxed PGC1α allele (PGC1α^fl^; JAX 009666) and those with muscle-specific expression of Cre recombinase under the human skeletal actin promoter (HSA-Cre; JAX 006149). The generation and initial characterization of genetically modified mouse lines used in this study were described previously^43–45^. Mice were genotyped by PCR using the following primers:

*MCK-PGC1*α *for* 5′-GCA GGA TCA CAT AGG CAG GAT GTG GCC-3′

*MCK-PGC1*α *rev* 5′-GGA AGA TCT GGG CAA AGA GGC TGG TCC-3′

*PGC1*α^*fl*^ *for* 5′-TCC AGT AGG CAG AGA TTT ATG AC-3′

*PGC1*α^*fl*^ *rev* 5′-TGT CTG GTT TGA CAA TCT GCT AGG TC-3′

*Cre* for 5′-GCG GTC TGG CAG TAA AAA CTA TC-3′

*Cre* rev 5′-GTG AAA CAG CAT TGC TGT CAC TT-3′.

### Tissue preparation

Animals examined in this study were euthanized by intraperitoneal injection of 0.05 ml Euthasol (Virbac Animal Health). For fluorescence imaging of muscle whole mounts, euthanized animals were transcardially perfused with PBS, pH 7.4. The sternomastoid, triangularis sterni, extensor digitorum longus (EDL), plantaris and soleus muscles were dissected and fixed in 4% phosphate-buffered paraformaldehyde, pH 7.4 for 20 minutes at room temperature and rinsed in three changes, 5 min. each, of PBS.

For muscle fiber type determination, fixed sternomastoid, EDL and soleus muscles were and frozen in Tissue-Tek Optimal Cutting Temperature compound (Sakura Finetek, Torrance, CA) with liquid nitrogen-cooled isopentane. The blocks of muscles were cut on a cryostat (Leica Biosystems, Buffalo Grove, IL) to produce 12-µm sections at an angle perpendicular to the long axis of the muscle fibers.

### Fluorescence immunohistochemistry

**Figure 1:**
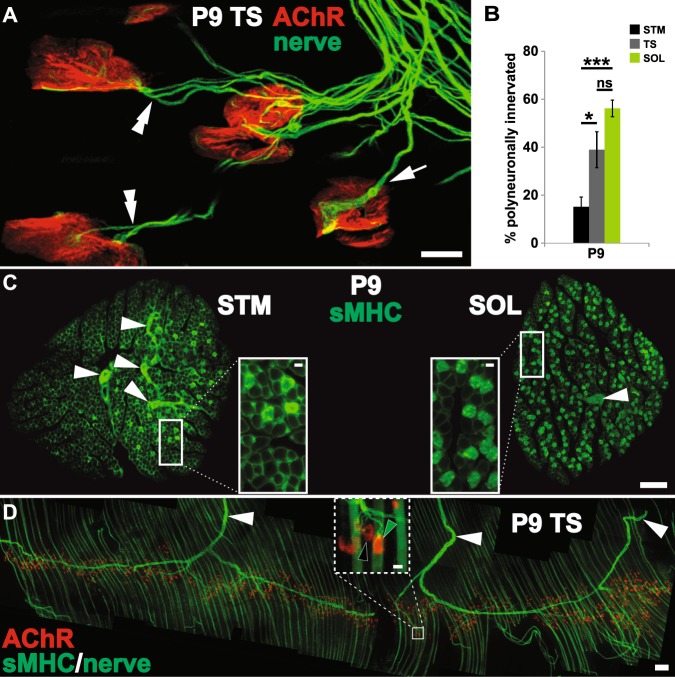
Muscle position along anterior-posterior body axis does not fully account for the timing of developmental neuromuscular synapse elimination. All data are from P9 mouse pups. (**A**) Innervation of NMJs in a triangularis sterni (TS), a mouse respiratory muscle. Despite NMJs that have completed synapse elimination and are each innervated by single motor axons (arrow), a significant fraction of NMJs remain polyneuronally innervated (double arrowhead). (**B**) The proportion of polyneuronally innervated NMJs in TS (~39%), bears a closer resemblance to that of a hindleg muscle, soleus (SOL; ~55%) than to a neck muscle, sternomastoid (STM; ~15%). (*p < 0.05, ***p < 0.001, ns = no statistical difference between SOL and TS) (**C**) Cross-sections of wildtype STM and SOL were immunolabeled for slow-twitch muscle fiber-specific antigen, slow MHC (MHCs). Distinct from the type I fibers (insets), the intramuscular nerve branches exhibit non-specific labeling (white arrowheads). Slow (type I) fibers make up only a small fraction of STM while a large fraction of SOL muscle fibers is of type I identity. (**D**) A whole-mount immunofluorescence labeling of mouse TS muscle for MHCs demonstrates the distribution of type I muscle fibers (green stripes running vertically in the montage). A large number of type I fibers appear evenly distributed throughout TS. The muscle was additionally labeled with α-BTX to mark the postsynaptic AChR aggregation, and an antibody against neurofilament (NF) to identify innervating axons (arrowheads). The inset is a higher magnification view of the boxed area in D that shows the striated labeling of MHCs in type I fibers. Green and black arrowheads indicate NMJs situated on type I and II (unlabeled) fibers, respectively. (Scale bars: 100 µm in **C** and **D**; 10 µm in A as well as in insets of **C** and **D**.)

To label surface nicotinic AChR at NMJs, fixed muscles were incubated with α-bungarotoxin (α-BTX, a snake toxin that binds specifically and with high affinity to AChR) conjugated to spectral variants of Alexa Fluor fluorescent dyes (AF-555 and AF-647; Invitrogen, Carlsbad, CA) prior to permeabilization (0.1 mg/ml). The motor axons were labeled with a monoclonal anti-neurofilament antibody (2H3; Developmental Studies Hybridoma Bank, University of Iowa, Iowa City, IA). If an NMJ is innervated by a single motor axon (Fig. 1A arrow), the synapse was considered as singly innervated NMJ. When an NMJ is innervated by 2 or more distinct motor axons (Fig. 1A double arrowheads), it was considered polyneuronally innervated. The stages of AChR aggregate maturation were identified as previously noted^46–48^. Briefly, the initially ovoid plaques of AChR aggregates morph successively into perforated plaques and open configurations.

Type I muscle fibers were labeled with one of two monoclonal anti-slow myosin heavy chain antibodies: anti-MHCs (Leica Biosystems), and A4.840 (Developmental Studies Hybridoma Bank). The specificity of the Leica anti-MHCs monoclonal (IgG1 isotype; 1:100 dilution) was verified by co-labeling of muscle cross-sections with A4.840 (IgM isotype; applied neat) and isotype-specific anti-mouse secondary antibodies conjugated to spectrally distinct fluorescent dyes (Fig. S1). A rabbit antibody against actinin α3 (actn3; Abcam, Cambridge, UK; 1:1000 dilution) labels all muscle fibers (Fig. S2) and was used to determine the total number of muscle fibers within a muscle cross-section. 4′,6-Diamidino-2-phenylindole (DAPI; 0.5 µg/ml) was used to identify nuclei. For a muscle cross-section, type I (slow) muscle fiber composition was determined as a fraction of all (actn3-positive) muscle fibers.

Images were acquired using a Zeiss LSM 780 confocal system (Image Analysis Laboratory, Texas A&M University) or a Leica DMR epifluorescence microscope equipped with a Hamamatsu cooled CCD camera. Confocal images were acquired using a 40X oil-immersion objective (N/A 1.4) with a 212.55 µm × 212.55 µm field of view at 2048 × 2048 pixel-density and 0.3 µm steps in the Z-axis. Analysis of digital images and determination of single- vs. polyneuronal innervation were performed using FIJI software^49^.

### Statistical analysis

Statistical analyses – one-way analysis of variance (ANOVA) with Bonferroni post hoc, linear regression, Student’s t-test, and Pearson’s/Spearman correlation coefficients – of raw data and the generation of histograms were performed using GraphPad Prism software (GraphPad; La Jolla, CA) and Excel spreadsheet software (Microsoft; Redmond, WA): *p < 0.05; **p < 0.01; ***p < 0.001. Numerical data are reported as mean ± standard error of means (SEM).

## Results

Progressive development along the anterior-posterior (AP or rostrocaudal) axis occurs for many aspects of mammalian neuromuscular development, including initial differentiation of motor neurons and muscles and the generation of functional neuromuscular connections and reflex circuits^50,51^. The timing of developmental synapse elimination in various skeletal muscles also seems to generally follow this AP developmental gradient^52^. Consistent with this observation, I have previously observed that the completion of neuromuscular synapse elimination in a neck muscle, the sternomastoid (STM), occurs several days prior to that in a hindlimb muscle, the soleus (SOL)^20,53^. Those results are confirmed in the present study. Additional influences independent of the AP axis, nevertheless, exert influence on the removal of redundant motor inputs^14,16,17,19,20,53–59^.

### Fiber type compositions of target muscles influence the timing of developmental synapse elimination

I first compared the degree of polyneuronal innervation among three different muscles located at distinct positions along the AP axis including SOL, STM and triangularis sterni (TS, a muscle found on the inner wall of the ribcage^60^). Counter to the AP axis-based prediction, I found that roughly half of the NMJs – identified with fluorescently-labeled α-bungarotoxin (α-BTX) – in SOL and TS are innervated by multiple axons at postnatal day (P) 9, while this was true for only ~15% of STM NMJs (Fig. 1A,B; SOL: 55.28 ± 2.92%, TS: 38.97 ± 7.50%, STM: 15.15 ± 4.09%; n ≥ 4, ≥58 NMJs per animal; p < 0.001, one-way ANOVA with Bonferroni post hoc vs SOL: p = 0.1674 for TS, p < 0.001 for STM, and p < 0.05 for TS vs STM). The proportions of NMJs that were polyneuronally innervated did not differ significantly between SOL and TS muscles at P9 (Fig. 1B). In addition to their relative positions along the AP axis, sternomastoid and soleus muscles differ significantly in their muscle fiber type composition (Fig. 1C): early in the second week of postnatal life, slow-twitch fibers make up a significant portion (31.14 ± 0.81%, n = 5) of SOL but only a small fraction of STM (3.70 ± 0.05%, n = 3; p < 0.0001, two-tailed t-test; see also^53^). The thinness of TS proved prohibitive in determining the slow muscle fiber contribution to its makeup via production of cross-sections. Based on whole-mount images (Fig. 1D), however, TS muscle fiber type composition appears to more closely resemble that of SOL rather than STM. The placement of muscles along the AP axis, thus, does not fully account for the intermuscular difference in the timing of neuromuscular synapse elimination as indicated by the fraction of NMJs still receiving multiple motor axon inputs at P9. Additionally, my examination of the TS muscle suggests that myofiber type composition may contribute to the difference in timing. I, therefore, examined whether the timing of synapse elimination differs between muscle fiber types.

**Figure 2:**
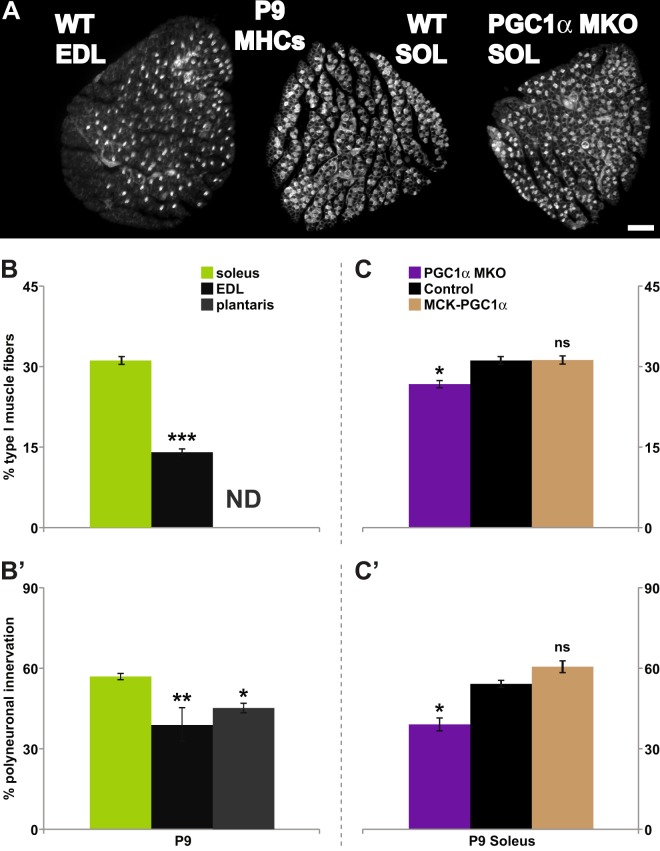
Fiber type composition of target muscles influences the timing of developmental synapse elimination. All data are from P9 mouse pups. (**A–C**) Transverse sections of control extensor digitorum lognus (**EDL**) and soleus (**SOL**) muscles from wildtype animals, as well as soleus muscle of muscle-specific knock-out and overexpression of PGC1α (**PGC1**α**-MKO**, and **MCK-PGC1**α, respectively); transgenic mice were stained for slow MHC isoform (**A**, MCK-PGC1α SOL shown). Control EDL, a “fast” muscle, consistently contained a smaller proportion of type I muscle fibers (~14%) compared to control soleus (~31%), a “slow” muscle (**B**). Type I muscle fiber contribution to plantaris muscle was not determined (ND). For the SOL muscle, PGC1α MKO (~26%), but not of MCK- PGC1α (~31%), differed from control SOL muscles (**C**). In control pups, ~56% of SOL NMJs were polyneuronally innervated compared to ~35% and ~45% of NMJs in EDL and plantaris, respectively (**B’**). Soleus of PGC1α MKO pups had a smaller fraction of polyneuronally innervated NMJs compared to controls (~39% vs. ~54%). No significant change was observed in the soleus of MCK-PGC1α (~60%). Thus, muscle fiber-specific genetic manipulation that decreases type I muscle fibers (PGC1α MKO) also alters the timing of developmental synapse elimination. (*p < 0.05, **p < 0.01, ***p < 0.001).

To control for the influence of AP positioning of the muscles examined, I compared SOL (rich in slow-twitch myofibers) with two fast twitch-rich hindlimb muscles extensor digitorum longus (EDL) and plantaris (PLT)^61,62^ situated in close proximity along the AP as well as the proximal/distal axes. My examination of transverse sections confirmed that EDL muscle has a significantly smaller fraction of constituent slow fibers than SOL (14.04 ± 0.63% vs. 31.14 ± 0.81% n ≥ 4 at P9; p < 0.0001, two-tailed t-test; Fig. 2A,B). I was, however, not able to confirm firsthand the reported low slow muscle fiber contribution to the makeup of PLT muscle^61,62^ owing to its tendon arrangement preventing production of cross-sections that include all muscle fibers. Consistent with the idea that constituent muscle fiber type composition influences the rate of neuromuscular synapse elimination, EDL and PLT consistently contained smaller fractions of polyneuronally innervated NMJs when compared to SOL at P9 (33.71 ± 4.75% for EDL, 45.14 ± 1.18% for PLT, 55.85 ± 1.16% for SOL, n ≥ 5, ≥59 NMJs per animal; p < 0.0001, one-way ANOVA with Bonferroni post hoc vs. SOL: p < 0.0001 for EDL and <0.01 for PLT; Fig. 2B’). These observations are consistent with a recent report by Personius and colleagues in which a smaller fraction of EDL NMJs are polyneuronally innervated compared to those in SOL at P8^56^ (but differ from a pair of earlier examinations^47,52^). Lastly, the present findings appear consistent with another previous study in which delayed synapse elimination is concurrent with significantly fewer type II fibers in muscles of a spinal muscular atrophy mouse model, SMAΔ7^53^. Together, these data support the possibility that muscles with greater fractions of slow fibers (SOL, TS) are slower to undergo synapse elimination versus muscles composed primarily of fast fibers (EDL, PLT, STM).

Next, I tested whether experimentally manipulating fiber type composition of a muscle would correspondingly affect the timing of synapse elimination. Soleus muscle offers several advantages for examining the potential influence of muscle fiber type on developmental synapse elimination. Firstly, it has significant proportions of both slow and fast fibers (Figs 1C, 2A,B) – thus, both fiber types can be readily sampled (see below for the intramuscular comparison of NMJs). Secondly, approximately 50–60% of the soleus motor endplates are polyneuronally innervated at P9: one can readily detect the deviation in the time course of synapse elimination in either direction. Additionally, alterations to its fiber composition are possible through genetic manipulation.

More specifically, I took advantage of mouse lines whose muscle-specific changes in the levels of the transcriptional co-activator peroxisome proliferator-activated receptor gamma coactivator 1- α (PGC1α) produce altered muscle fiber type compositions. First, I utilized mutant mice that harbor myofiber-specific inactivation of PGC1α (PGC1α-MKO); this mouse line exhibits a reduced proportion of constituent slow muscle fibers^43^. Second, transgenic mice that overexpress PGC1α under a myofiber-specific promoter (MCK-PGC1α) have been reported to exhibit increased proportions of slow fibers^44^. I confirmed a modest – yet consistent – reduction in slow fibers in the soleus muscles of PGC1α-MKO animals, but failed to detect an increase in slow fiber frequency in SOL of MCK-PGC1α transgenic mice (26.7 ± 0.7% for PGC1α-MKO, 31.1 ± 0.8% for controls, 31.2 ± 0.8% for MCK-PGC1α, n ≥ 3, p < 0.01, one-way ANOVA with Bonferroni post hoc vs. controls: p < 0.05 for PGC1α-MKO and >0.99 for MCK-PGC1α; Fig. 2A,C). The apparent inability of the MCK-PGC1α transgene to drive PGC1α overexpression in SOL^44,63^ likely underlies the absence of any detectable increase in slow fibers in the MCK-PGC1α transgenic SOL.

We then asked whether the time course of synapse elimination was altered in parallel with the changes in the abundance of slow fibers. The proportion of SOL NMJs polyneuronally innervated at P9 did not differ between the two control groups: wildtype pups (for MCK-PGC1α; 52.39 ± 2.14% n = 5) and those homozygous for the floxed PGC1α allele (PGC1α^fl/fl^; 56.37 ± 0.38%, n = 4, p = 0.1476). The values for the two control groups (wildtype and PGC1α^fl/fl^) were pooled for further statistical analysis. At P9, PGC1α-MKO SOL had ~15% fewer polyneuronally innervated NMJs compared to those of control pups while no difference was seen between control and MCK-PGC1α (39.1 ± 2.4% for PGC1α-MKO, 54.2 ± 1.3% for controls, 60.6 ± 2.2% for MCK-PGC1α, n = 4, ≥85 NMJs per animal; p < 0.0001, one-way ANOVA with Bonferroni post hoc vs. controls: p = 0.0001 for PGC1α-MKO and p = 0.0617 for MCK-PGC1α; Fig. 2C). The difference in the timing of synapse elimination originates not from any gross developmental differences in transgenic animals (Fig. S3): the average weight of the animals tested did not differ between groups (controls vs. PGC1α MKO-vs. MCK-PGC1α: 4.79 ± 0.23 g vs. 5.30 ± 0.33 g vs. 4.6 ± 0.49 g, n ≥ 4; p = 0.3989, one-way ANOVA) nor was the degree of polyneuronal innervation impacted by the presence of floxed PGC1α alleles (wildtype vs. PGC1α^fl/fl^; 52.39 ± 2.14% vs. 56.37 ± 0.38% at P9, n ≥ 4, p = 0.1476) or muscle-specific expression of the Cre-recombinase (wildtype vs. HSA-Cre; 45.01 ± 2.82% vs. 47.33 ± 0.88% at P10, n = 3, p = 0.4766). A decrease in physical activity and muscle function were reported for PGC1α MKO mice^43^. It is, however, unclear whether these changes stem from changes to muscle physiology and/or motor neuron activity. Even if the decrease in physical activity were due to changes in motor neuron activity, the overall decrease in neuromuscular activity is predicted to delay the timing of synapse elimination^14,64^, the opposite of what was observed with the PGC1α muscle knock-out pups (Fig. 2C’). As the change to SOL fiber type composition results from the use of a myofiber-specific promoter, these findings, thus, demonstrate that the fiber type compositions of target muscles influence the competition amongst converging motor inputs myofibers initially receive.

**Figure 3:**
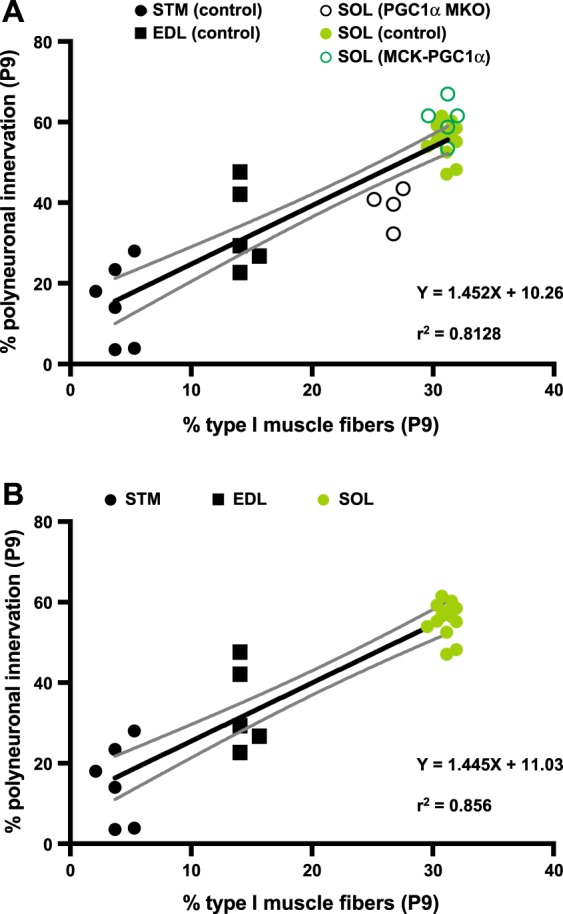
A linear regression of the frequency of polyneuronally innervated NMJs on the abundance of type I muscle fibers. (**A**) The fraction of NMJs that remain polyneuronally innervated at P9 for control muscles – EDL (n = 5), SOL (n = 14), STM (n = 6) – as well as PGC1α MKO SOL (n = 4) and MCK-PGC1α SOL (n = 5) were regressed on the mean type I muscle fiber frequency for each muscle. The analysis revealed a close relationship (r^2^ = 0.8128) with the best-fit line slope (1.452 ± 0.1232; solid black line) that deviates significantly from zero (p < 0.0001). (**B**) An identical analysis using only the control EDL, SOL and STM muscles similarly revealed a close relationship (r^2^ = 0.856) with the best-fit line slope (1.445 ± 0.1236; solid black line) that deviates significantly from zero (p < 0.0001). The confidence bands (the two curved gray lines) are 95% sure to enclose the true best-fit linear regression line.

A linear regression analysis was performed as an attempt to determine whether a meaningful relationship exists between the proportion of polyneuronally innervated NMJs and the percentages of the slow fibers in the respective muscles (EDL, SOL and STM as well as PGC1α-MKO and MCK-PGC1α SOL) using values already at hand (Figs 1, 2). The analysis demonstrated a close relationship (r^2^ = 0.8128) where the slope of the best-fit line (1.452 ± 0.123) deviated significantly from zero (p < 0.0001; Fig. 3A). An identical analysis of only the control muscles (EDL, SOL and STM) similarly demonstrated a strong correlative relationship between the abundance of slow fibers and the fraction of NMJs that remain innervated by multiple motor axons (r^2^ = 0.856; best-fit slope of 1.445 ± 0.123, p < 0.0001; Fig. 3B). Thus, the present experimental findings, when subjected to statistical scrutiny, suggest that the differences in slow myofiber contribution likely account for a significant fraction of the variation in the timing of synapse elimination observed amongst muscles examined.

### Target muscle fibers influence the resolution of local synapse elimination

**Figure 4:**
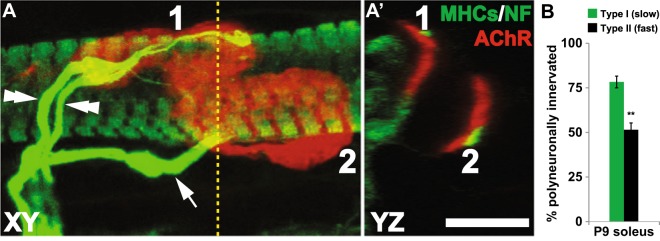
Delayed resolution of synapse elimination for NMJs situated on type I muscle fibers. (**A**) Neurofilament (**NF**, for axons) and postsynaptic **AChR** are labeled to determine the number of the axons that innervate individual NMJs. Muscle fibers were also labeled for slow MHC (**MHCs**) to determine the fiber type identity of the target fiber on which synapses were situated. To the left is a maximum intensity projection of a confocal stack containing 2 P9 NMJs. NMJ1 is innervated by two axons (double arrowheads) while NMJ2 is singly innervated (arrow). Virtual cross-section of the confocal stack (**A’**), taken at the position indicated by the vertical dotted line, demonstrates that NMJ1 resides on a type I muscle fiber, while NMJ2 is on a type II fiber. (**B**) A significantly greater fraction of NMJs situated on type I fibers remain polyneuronally innervated compared to those on type II fibers (**p < 0.01). (Scale bar: 10 µm.)

The above findings also raise the possibility that developmental synapse elimination occurs at different rates for NMJs situated on fibers with distinct contractile properties. It is, however, equally possible that altered fiber type compositions alter the milieu of a given muscle as a whole, within which the elimination of surplus motoneuron inputs on all synapses are similarly influenced. In an attempt to distinguish between the two possibilities, I compared the proportions of SOL NMJs situated on slow and fast fibers that receive multiple innervations. To determine unambiguously the fiber type associated with each NMJ, I employed confocal microscopy that allows examination of each synapse in all three spatial dimensions (X, Y and Z; Fig. 4A). Examination of the NMJs revealed that 78.3 ± 3.3% of slow fibers remain polyneuronally innervated at P9 vs. 51.5 ± 3.8% of fast fibers (Fig. 4B; n = 4, 58–295 NMJs per animal; p < 0.01, unpaired t-test). The intramuscular comparison of SOL NMJs indicates that synapse elimination is delayed for motor endplates of slow muscle fibers compared to fast fibers. Such intramuscular comparison of muscle fiber types within SOL clearly demonstrates local differences in the timing of synapse elimination between fiber types, which subsequently predicts that a similar difference between fast and slow myofibers exists also in other muscles – including EDL, plantaris, sternomastoid and triangularis sterni.

### Maturation of postsynaptic AChR aggregates does not influence developmental synapse elimination

Aspects of the postsynaptic muscle fiber that differ between slow and fast types and, subsequently, may influence local synapse elimination remain undefined. One structural aspect I considered was the postsynaptic motor endplate – the substrate for the competition amongst convergent motor inputs – and its morphology. The competition ensues within the confines of the AChR aggregates^21,65^, which initially resemble ovoid plaques but transition eventually into shapes that resemble “pretzels” as the synapse matures over the first three postnatal weeks, coincident with neuromuscular synapse elimination^46–48,53,65,66^. The arborisation of motor axon terminals dictates the changes in the shape of the postsynaptic AChR aggregates *in vivo*: immature postsynaptic AChR aggregates of denervated muscles fail to mature^66^ and even disperse^67^. The plaque-to-pretzel transition of AChR aggregates, however, can occur *in vitro* in complete absence of motor axon influence^46^ and suggests that motor endplate may reciprocally influence the branching of motor axon terminals. The turnover and/or removal of AChR within the AChR-rich plaques and the concurrent changes in the postsynaptic landscape, thus, may have consequences for the timing of developmental synapse elimination. Genetic manipulations that either accelerate or delay synapse elimination also produce corresponding changes in the rate of postsynaptic maturation^53,58^.

**Figure 5:**
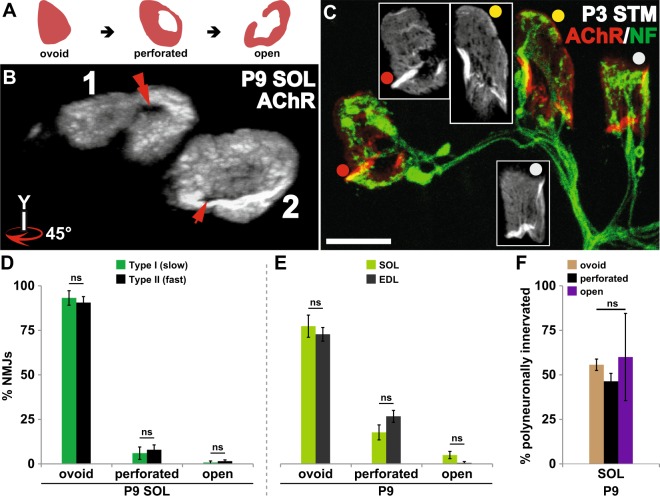
Maturation of postsynaptic AChR aggregates does not influence the timing of synapse elimination. (**A**) The cartoon illustrates the progressive morphological maturation of postsynaptic AChR aggregates at developing NMJs. (**B**) The AChR aggregates of the two P9 soleus (SOL) NMJs in Fig. 4A are shown with a clockwise 45° rotation about the y-axis to better visualize the perforations present within each of the AChR aggregates. The innervation state of the developing synapse and the maturity of the postsynaptic morphology do not bear a correlative relationship: the dually-innervated NMJ1 (Fig. 4A) is associated with a well-defined perforation within its AChR aggregate, while the NMJ2 – having completed synapse elimination – has an immature postsynaptic assembly that is only beginning to form a perforation in its AChR aggregate. (**C**) The postsynaptic AChR aggregates of the three polyneuronally innervated P3 sternomastoid (STM) NMJs show varying degrees of maturation. Of the three AChR aggregates shown, one has developed into an open configuration (red dot), one remains an ovoid plaque (grey dot), and another has started to develop perforations (yellow dot). (**D**,**E**) The maturity of postsynaptic morphology does not differ between type I and II fibers nor between soleus (a “slow” muscle) and EDL (a “fast” muscle). (**F**) Similar proportions of NMJs are polyneuronally innervated regardless of the postsynaptic morphology – ovoid, perforated or open plaques. (ns = no statistical difference between groups) (Scale bar: 10 µm).

The maturation of postsynaptic AChR-rich plaques (Fig. 5A; scored 1 for ovoid, 2 for perforated, and 3 for open or “C”-shaped), however, bears no significant correlation with the loss of redundant motor axons that innervate each individual motor endplate (Spearman r = −0.0146, p = 0.7060; 670 NMJs from 4 P9 wildtype pups; also see Figs 4A and 5B). Consistent with this, my examination of STM NMJs at P3, in which virtually all NMJs remain innervated by multiple motor axons^20^, revealed the varying degree of maturation undertaken by the postsynaptic AChR aggregates, providing more evidence that postsynaptic AChR morphology is causally unrelated to the removal of excess motor inputs at developing endplates. An example is shown in Fig. 5C: all three NMJs are polyneuronally innervated despite the differences in the morphology of the postsynaptic AChR aggregates. Nor does the maturity of AChR aggregate morphology differ between type I and II fibers within SOL (Fig. 5D) or between type I- or type II-rich muscles - SOL and EDL (Fig. 5E; see also^47^). Most importantly, the AChR maturation score bears little correlation to the fiber type of the myofiber on which they are found (Spearman r = 0.0155, p = 0.6895; 670 NMJs and their parent muscle fibers from 4 P9 wildtype pups) and the proportions of polyneuronally innervated NMJs at different stages of postsynaptic maturation do not significantly differ (ovoid vs. perforated vs. open: 55.7 ± 3.2% vs. 46.3 ± 4.5% vs. 60.0 ± 24.49, n = 4, p = 0.797, one-way ANOVA with Bonferroni post hoc comparison; Fig. 5F). My observations are consistent with reported manipulations that alter the postsynaptic morphology or its molecular component without any consequences for the timing of developmental synapse elimination. Synapse elimination preceded normally in mutants with increased AChR turnover and compromised postsynaptic maturation^68^ or increased AChR-rich area^69^. Conversely, mature “pretzel”-shaped AChR aggregates develop for a small number of polyneuronally innervated NMJs that persist in adult mice lacking MHC1 molecules^70^. I conclude, therefore, that changes in the morphology of postsynaptic specialization are a consequence of developmental alterations to the presynaptic morphology^66,67^ and unlikely to be a means by which the postsynaptic muscle fibers influence the removal of redundant axonal inputs.

## Discussion

The regulation of developmental synapse elimination at NMJs is multifactorial. At the cellular level, activities of synaptic glial cells (tSCs) and converging motor axon terminals are known to affect the timing and/or the winner of this competition^14–17,19,20,71^. The potential influence of the postsynaptic muscle fibers, however, remained ambiguous. My present findings provide several lines of evidence that properties of postsynaptic muscle fibers influence the competition among the axons that provide innervation. Firstly, while the timing of synapse elimination for the muscles examined generally agrees with the AP gradient in development^52^, present findings demonstrate that intermuscular difference in the timing of synapse elimination is influenced also by the relative composition of the constituent muscle fiber types. The muscles with a greater proportion of fast fibers consistently had a smaller fraction of NMJs innervated by multiple motor axons. Secondly, a genetic manipulation known to reduce the relative contribution of slow fibers also accelerates the time course of synapse elimination consistent with the idea that synapse elimination is retarded in slow fiber-rich muscles. Lastly, an intramuscular comparison revealed that synapse elimination is significantly delayed on slow compared to fast fibers within the soleus muscle. While the cellular and molecular mechanisms that contribute to the differences in the timing of redundant motor input pruning remain undefined, my results strongly suggest that the influence of divergent muscle fiber properties on synapse elimination occurs locally. Early studies comparing synapse elimination of twitch fiber types, however, failed to highlight any differences in their timing of synapse elimination^47,52,72,73^. The reasons for the discrepancy in observations – despite the differences in the species examined and in the experimental approaches – remain unclear.

The present findings, taken together with previous reports, may suggest that the rates of synapse elimination indeed differ between muscles and between fiber types. Firstly, I have previously reported that while virtually all NMJs of STM and SOL are polyneuronally innervated 3 days after birth, a progressively smaller fraction of STM NMJs compared to SOL remain polyneuronally innervated through postnatal days 6 and 9^20^. Secondly, while virtually all SOL NMJs are polyneuronally innervated at P3, the fractions of NMJs still polyneuronally innervated at P9 differ between types I and II muscle fibers. A recent report^7^ demonstrated that motor axons initially establish synaptic connections, albeit weak, with nearly all available NMJs within a given target muscle. This observation suggests that the number of axons that initially converge at a typical neonatal endplate does not differ between types I and II muscle fibers. Together, these findings suggest that the differences in the timing of resolution of synapse elimination between muscles and distinct fiber types within a given muscle both likely stem, at least partly, from differences in the rate of synapse elimination of distinct fiber types.

In addition to the progression of differentiation down the AP axis, there are developmental programs that appear to influence the generation of the neuromuscular system and potentially impact the timing of synapse elimination. Muscle fibers generated during the first wave of myogenesis are reported to be primarily of slow contracting type while a majority of those formed during secondary myogenesis become fast contracting^74^. Moreover, skeletal muscles could be assigned either “Fast” or “Delayed” synapsing with respect to the initial formation of NMJs and subsequent synaptic stability upon denervation^75^. Had synapse elimination been completed in the order in which the muscle fibers are generated, slow fibers would be expected to reach single innervation before fast fibers. Similarly, if the order in which NMJs form dictates the timing of redundant motor input pruning, the expected outcome where “Fast” synapsing muscles – including EDL – outpace “Delayed” synapsing muscles (PLT and SOL) would deviate from my present observations. The results of this study, in contrast, strongly favor the idea that properties of individual myofibers, perhaps more so than the order of generation of a fiber or its resident synapse, influence the developmental pruning of surplus motor inputs. Indeed, a linear regression analysis of my present data suggests that differences in the abundance of slow fibers account for a significant fraction of the variability in the timing of synapse elimination amongst the muscles examined.

The patterns in which muscles are stimulated to contract is known to influence the time course of synapse elimination. A direct muscle stimulation with a pattern said to mimic the activity pattern of “fast” motor neurons accelerates, by 2–3 days, the developmental synapse elimination in rat SOL^17^. Such stimulation also produced a shift in contractile properties of SOL – a “slow” muscle – to resemble a “fast” muscle including more rapid rise times for isometric twitches, a requirement of higher stimulation frequency for fusion of twitches, and larger tetanus contractions despite smaller isometric twitches. While such direct stimulation may also activate preterminal axons or neuromuscular junctions in addition to the muscle fibers, the concurrent changes in contractile properties consistent with slow-to-fast fiber type conversion and precocious synapse elimination are consistent with my current findings where removal of excess motor inputs lags on slow type muscle fibers. Retention of multiple innervation by tonic muscle fibers – those that do not generate action potentials – in reptiles^30,31^ may also suggest that properties of target muscle fibers can influence synapse elimination. The observed difference in the timing of synapse elimination between twitch fiber types is, then, perhaps not unexpected.

A motor neuron and the set of muscle fibers it innervates compose a motor unit. Within a mature mammalian motor unit, all the muscle fibers share a common fiber type^76^. Early studies suggest selective innervation of different fiber types, each by motor neurons with an appropriate activity pattern^77–79^. Other studies, however, demonstrated a significant degree of mismatches during early postnatal development^80,81^ that would be expected following pervasive innervation of target muscles during early development^7^. Such findings suggest that refinement of neuromuscular connections during postnatal maturation would necessitate preferential pruning of inputs with inappropriate activity patterns. Both scenarios are compatible with the current observation where the resolution of synapse elimination on distinct fiber types is temporally offset. Interestingly, a recent study indicates that “fast/slow identity” of postsynaptic muscle fibers can influence that of the innervating motor neurons^80^. This may imply that any significant changes to the sizes of motor units (both fast and slow) at the peak of polyneuronal innervation – which could indirectly alter the timing of synapse elimination – need not occur. This observation, on the other hand, suggests that it is just as possible for muscle fibers to influence synapse elimination indirectly via alterations of partnering motor axons as it is to do so directly.

The mechanism(s) by which appropriate matching of pre- and postsynaptic partners is achieved remains obscure. Even if the developmental scenario requires that motor neurons seek out appropriate target fiber types for initial innervation during initial synapse formation, such ability for the motor axon and/or the target muscle fiber to recognize the correct synaptic partner would be necessary. Whether direct or indirect, muscle fibers must be able to discriminate between appropriate and inappropriate motor inputs or provide the motor neurons the means to do so. A repulsive axon guidance molecule ephrin-A3, recently found to be expressed specifically in slow fibers postnatally^82^, may actively repel immature axons of “fast” motor neurons. Class 1 major histocompatibility complex (MHC1) molecules, whose genetic inactivation leads to a delay in synapse elimination, both at the NMJs^70^ as well as the relay cells of the visual system within the lateral geniculate nucleus^83^, also provide a family of attractive candidates that may be involved in recognition and removal of non-compatible synaptic partners. Protracted synapse elimination found in neural cell adhesion molecule (NCAM) deficient animals, however, appears to stem indirectly from altered presynaptic activity^57^. On the other hand, the reported upregulation of NCAM by denervated muscle fibers^84^ may be interpreted as an attempt to provide a more permissive substrate for any axons that may subsequently come to innervate them regardless of the downstream mechanism. Secreted factors – such as brain-derived neurotrophic factor (BDNF), whose expression and functional maturation is regulated by activity^55,85^ or fibroblast growth factor binding protein 1 (FGFBP1)^86^ – present another potential avenue through which distinct muscle fibers may influence local synaptic competition. It is presently unknown whether distinct muscle fiber types differentially express BDNF, FGFBP1, MHC1 or NCAM. Additionally, presumed differences in activity levels of motor neurons that innervate slow and fast fibers may lead to differential expression of type-III neuregulin1^87^, a motor neuron-derived factor that influences the timing of synapse elimination through the activation of tSCs^20^.

In light of the present results, it is tempting also to speculate on whether the motor endplates of slow fibers are less vulnerable, compared to fast fibers, to age- and disease-induced denervations. Evidence of both a rearrangement of the distribution of muscle fibers within motor units as well as a prominent shift in the muscle contractile properties towards a slower, more fatigue-resistant characteristic in aged rodents and humans^88–95^ may suggest preferential denervation of fast fibers that transdifferentiate into slow fibers upon re-innervation by a slow motor axon. Several recent studies of amyotrophic lateral sclerosis (ALS)^96–101^, the disease etiology of which appears not to be motoneuron-autonomous^102,103^, provide additional support for this idea. NMJs of slow fiber-rich muscles – SOL and TS – display a delayed degeneration compared to fast fiber-rich muscles^99,104,105^. The relative sparing of these slow muscles to disease progression likely stems from the reported resistance to denervation of NMJs situated on slow fibers^96,98,101^. These findings may further suggest that, at least in muscles with significant slow motor neuron innervation, the two distinct pools of motor neurons – one compensatory pool that grows sprouts to occupy the endplates left vacant by the dying-back axons of the other pool^104^ – may partition with the contractile properties of their target muscle fibers. The observation that the integrity of NMJs found on slow fibers is relatively refractory to neuromuscular pathology appears not to be isolated to ALS. Muscle-specific overexpression of PGC1α, and therefore an increase in slow fibers, is also reported to alleviate the decline in the integrity of NMJs associated with sarcopenia^106^ and a mouse model of muscular dystrophy^107^. The present findings, in which the timing of synapse elimination was altered through muscle fiber-specific genetic manipulation, motivate a search for muscle-derived factors differentially expressed among fiber types that confer increased stability to neuromuscular synaptic connections. Such synapse stabilizing factors potentially represent a novel means to treat ALS and other neurodegenerative diseases with synaptic degeneration early in disease progression. Consistent with such therapeutic potential of synapse stabilizing factors, enhanced activation of muscle-specific kinase – necessary for both formation and maintenance of NMJs^108–110^ – either by genetic or pharmacological means – delays ALS disease progression in a mouse model^111,112^.

## Supplementary information

SUPPLEMENTARY INFO

## Data Availability

All data generated or analysed during this study are included in this published article and its Supplementary Information file.
