# Supplementary material for: Differences in the constituent fiber types contribute to the intermuscular variation in the timing of the developmental synapse elimination
Source: Sci Rep. 2019 Jun 18;9:8694. doi: 10.1038/s41598-019-45090-6 (PMC6582271; doi:10.1038/s41598-019-45090-6)
Supplement: Supplementary file 1 — SUPPLEMENTARY INFO [file 41598_2019_45090_MOESM1_ESM.pdf]

Supplementary Information

Differences in the constituent fiber types contribute to the intermuscular variation in the timing of the developmental synapse elimination

Young il Lee*
Department of Biology, Texas A&M University, College Station, TX 77843

***Correspondence**
Department of Biology
Texas A&M University
3474 TAMU
College Station, TX
77843
ylee@bio.tamu.edu

Figure S1. **Validation of a monoclonal antibody against slow MHC isoform.** A fresh frozen cross-section of an adult soleus muscle was co-labeled with two mouse monoclonal antibodies: A4.840 (IgM isotype) and another anti-slow MHC (MHCs; IgG1 isotype). The antibodies were visualized with isotype-specific anti-mouse secondary antibodies. Notice that both antibodies label the same set of muscle fibers (type I "slow"). (Scale bar: 10 μm)

Figure S2. **Determination of slow twitch myofiber contribution to skeletal muscle fiber composition.** Type I "slow" muscle fibers were labeled in muscle transverse sections with anti-slow MHC mouse monoclonal antibody (MHCs). The same sections were counter labeled with anti-actinin α3 (actn3) rabbit antibody that labels all twitch muscle fibers and a nuclear label (DAPI). The contribution of slow muscle fibers to the make-up of a muscle was expressed as a fraction of actn3 labeled muscle fibers. (Scale bar: 100 μm)

Figure S3. **Floxed PGC1α alleles or muscle-specific expression of Cre recombinase do not alter the growth of pups nor the timing of synapse neuromuscular synapse elimination.** (**A**) average weights of P9 pups whose PGC1α alleles were inactivated specifically in muscle (PGC1α MKO) and those with muscle-specific over-expression of PGC1α (MCK-PGC1α) did not differ from control P9 pups. (**B**&**C**) The degree to which soleus NMJs remain polyneuronally innervated was not influenced by the presence of floxed PGC1α alleles (without Cre-recombinase, at P10; **B**) nor in presence of muscle-specific expression of Cre recombinase (at P9, **C**).

Lee
Supplementary Figure 1

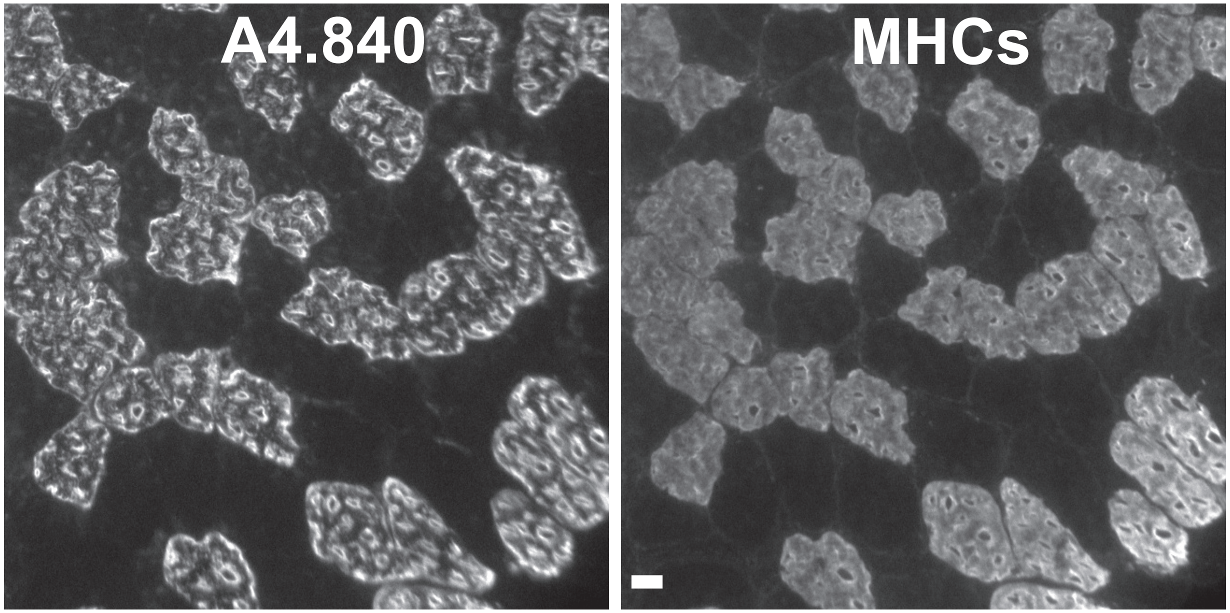

Figure S1. Validation of a monoclonal antibody against slow MHC isoform. A fresh-frozen cross-section of an adult soleus muscle was co-labeled with two mouse monoclonal antibodies: A4.840 (IgM isotype) and another anti-slow MHC (MHCs; IgG1 isotype). The antibodies were visualized with isotype-specific anti-mouse secondary antibodies. Notice that both antibodies label the same set of muscle fibers (type I “slow”). (Scale bar: 10 μm)

Lee
Supplementary Figure 2

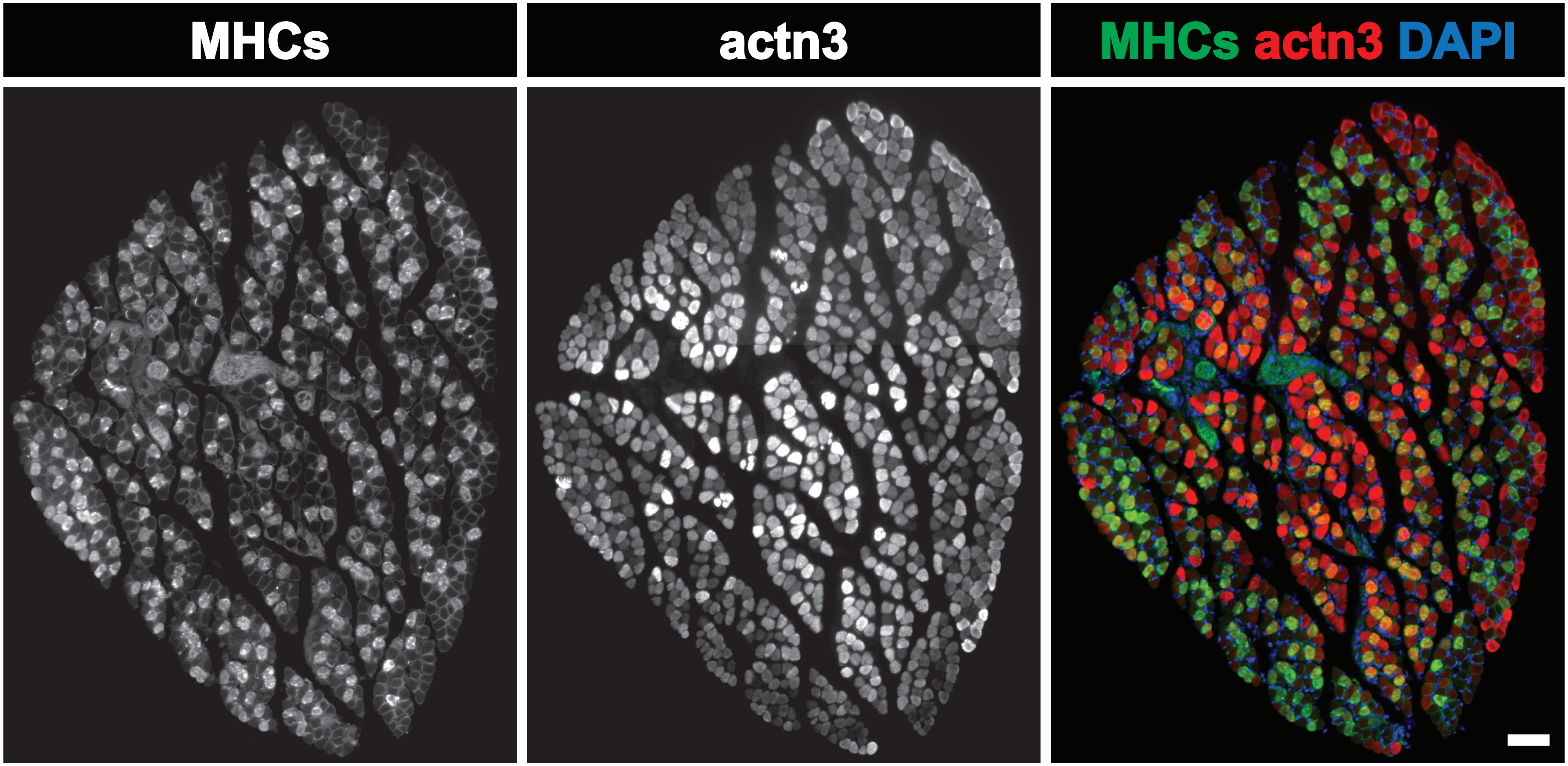

Figure S2. Determination of slow twitch myofiber contribution to skeletal muscle fiber composition. Type I "slow" muscle fibers were labeled in muscle transverse sections with anti-slow MHC mouse monoclonal antibody (MHCs). The same sections were counter labeled with anti-actinin α3 (actn3) rabbit antibody that labels all twitch muscle fibers (actn3) and a nuclear label (DAPI). The contribution of slow muscle fibers to the make-up of a muscle was expressed as a fraction of actn3 labeled muscle fibers. (Scale bar = 100 µm)

Lee
Supplementary Figure 3

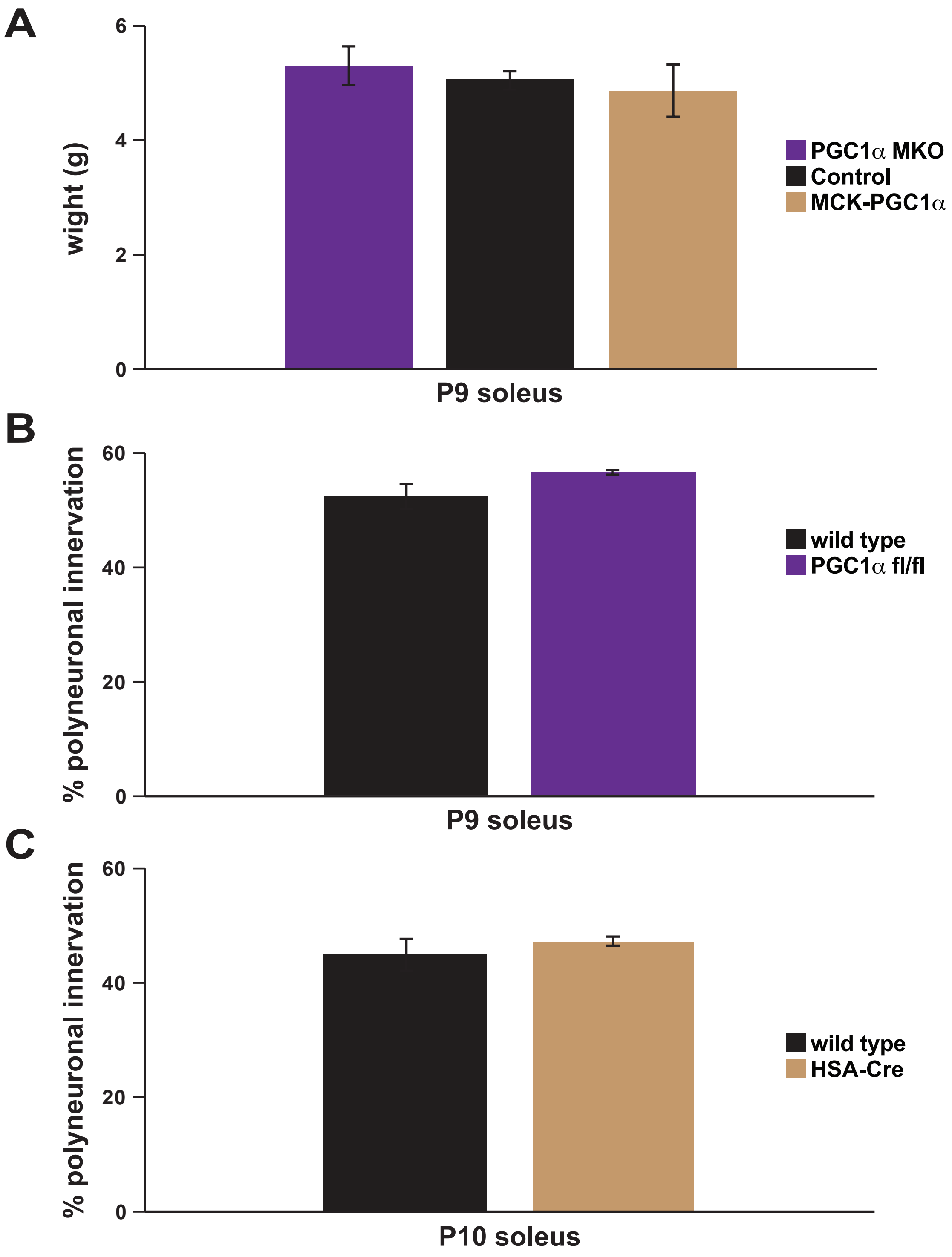

Figure S3. Floxed PGC1α alleles and muscle-specific expression of Cre recombinase does not alter the growth of pups nor the timing of synapse neuromuscular synapse elimination. (A) average weights of P9 pups whose PGC1α alleles were inactivated specifically in muscle (PGC1α MKO) and those with muscle-specific over-expression of PGC1α (MCK-PGC1α) did not differ from control P9 pups. (B&C) The degree to which soleus NMJs remain polyneuronally innervated was not influenced by the presence of floxed PGC1α alleles at P10 (without Cre-recombinase, at P10; B) nor in presence of muscle-specific expression of Cre recombinase (at P9, C).
